# Comparative associations of Anakinra and Tocilizumab initiation with in-hospital mortality in severe COVID-19: A single-center sequential cohort study

**DOI:** 10.1371/journal.pone.0357671

**Published:** 2026-09-02

**Authors:** Fevzi Necati Avsar, Nihat Kılıcaslan, Tuncay Sahutoglu

**Affiliations:** 1 Department of Internal Medicine, Sanliurfa Mehmet Akif Inan Training and Research Hospital, University of Health Sciences, Türkiye; 2 Department of Radiology, Sanliurfa Mehmet Akif Inan Training and Research Hospital, University of Health Sciences, Türkiye; 3 Division of Nephrology, Department of Internal Medicine, Sanliurfa Mehmet Akif Inan Training and Research Hospital, University of Health Sciences, Türkiye; Children’s National Hospital, George Washington University, UNITED STATES OF AMERICA

## Abstract

**Background:**

Interleukin-1 and interleukin-6 blockade have been widely used in severe COVID-19, but comparative real-world evidence regarding anakinra and tocilizumab in critically ill patients remains limited. We evaluated the associations of first initiation of anakinra or tocilizumab with 28-day and overall in-hospital mortality.

**Methods:**

This single-center retrospective ICU cohort included adults with severe COVID-19. During hospital days 2–10, daily actions were classified as anakinra initiation, tocilizumab initiation, or control/defer. Calibrated overlap-weighted modified-Poisson models estimated associations with 28-day and overall in-hospital mortality; ferritin-adjusted sensitivity analyses were performed.

**Results:**

Of 306 patients, 241 patients contributed 1,297 eligible person-days. Calibration met the prespecified balance criterion for the included covariates, although effective support for tocilizumab initiation remained limited. For 28-day in-hospital mortality, adjusted RRs versus control/defer were 0.99 (95% CI, 0.80–1.22) for anakinra and 0.99 (95% CI, 0.71–1.37) for tocilizumab. No clear associations were identified in the head-to-head comparison or for overall in-hospital mortality; ferritin-adjusted estimates were similar.

**Conclusions:**

In this retrospective ICU cohort, neither anakinra nor tocilizumab initiation showed a clear association with 28-day or overall in-hospital mortality. Estimates were imprecise, particularly for tocilizumab, and remain susceptible to residual confounding.

## Introduction

Coronavirus disease 2019 (COVID-19) exhibits a broad clinical spectrum ranging from asymptomatic infection to severe pneumonia and death [1]. In a subset of patients, disease progression is driven by a hyperinflammatory state characterized by cytopenia, hyperferritinemia, persistent fever, and acute respiratory distress syndrome (ARDS), commonly referred to as cytokine storm syndrome [2–5]. Autopsy studies of patients who died from SARS-CoV-2 infection have demonstrated that an exaggerated host immune response may culminate in a fulminant inflammatory cytokine cascade contributing to fatal outcomes [6].

Among the proinflammatory mediators implicated in severe COVID-19, interleukin-1 (IL-1) and interleukin-6 (IL-6) are important components of the dysregulated inflammatory response, although their relative contributions vary across clinical phenotypes and patient populations [5,7,8]. IL-1α is released early during tissue injury and infection and promotes downstream production of IL-6, tumor necrosis factor-α, granulocyte–macrophage colony-stimulating factor, and interleukin-17 [9]. Elevated IL-6 concentrations have been associated with severe adult COVID-19 and diffuse alveolar damage leading to acute respiratory distress syndrome [2,6,7]. In children, acute SARS-CoV-2 infection is generally milder, whereas multisystem inflammatory syndrome in children represents a distinct postinfectious hyperinflammatory phenotype in which IL-1, IL-6, and tumor necrosis factor-α may be elevated [10]. In patients with primary immunodeficiencies, impaired viral control and the nature of the underlying immune defect may produce heterogeneous inflammatory responses, including cytokine-release syndromes [11]. These population-specific differences limit direct extrapolation of cytokine profiles and responses to cytokine-targeted therapy among immunocompetent adults, children, and immunocompromised patients.

Targeted immunomodulatory therapies directed at these cytokines have therefore been investigated in the management of severe COVID-19. Tocilizumab is a recombinant monoclonal antibody directed against the IL-6 receptor, whereas anakinra is a recombinant human IL-1 receptor antagonist. Both agents were widely used during the pandemic in patients with severe inflammatory manifestations of COVID-19 [12,13]. However, real-world data evaluating their comparative effectiveness in critically ill patients admitted to the ICU remain limited, particularly in settings where treatment allocation is physician-driven rather than protocolized.

The distinctive contribution of this study is its use of exact calendar treatment dates to construct sequential daily risk sets in which anakinra initiation, tocilizumab initiation, and continued treatment deferral were evaluated as three distinct treatment decisions. This design was intended to preserve treatment timing, reduce immortal-time bias, and avoid combining two biologically distinct immunomodulators into a single exposure category.

In this single-center ICU-based observational cohort of hospitalized patients with severe COVID-19, we evaluated whether first initiation of anakinra or tocilizumab during hospital days 2–10 was associated with 28-day and overall in-hospital mortality after adjustment for baseline disease severity and other prespecified covariates. Using a sequential daily treatment-decision framework, we compared initiation of each agent with control/defer and with one another. We hypothesized that initiation of either cytokine-targeted therapy would be associated with lower mortality than control/defer.

## Methods

This single-center, retrospective observational study was conducted in the intensive care units (ICUs) of Şanlıurfa Mehmet Akif İnan Training and Research Hospital. The study population comprised adults admitted with COVID-19 pneumonia between August 2020 and February 2022. This interval represents the historical patient-admission period; the clinical information used in the study originated from care provided during these admissions. Data were subsequently extracted from the hospital electronic medical record system (FONET HBYS).

Eligible patients were aged 18 years or older, had confirmed SARS-CoV-2 infection by polymerase chain reaction testing, and demonstrated radiologic findings consistent with COVID-19 pneumonia on thoracic computed tomography (CT). All included patients required ICU admission due to acute respiratory failure. Patients were admitted either directly from the emergency department or transferred from inpatient wards following clinical deterioration.

Disease severity at ICU admission was assessed both radiologically and clinically. Thoracic CT scans obtained at admission were evaluated by a radiologist with more than ten years of experience. This scoring method, based on the extent, distribution, and pattern of pulmonary involvement, generates a total score ranging from 0 to 72 and was used to quantify radiologic severity [14]. Respiratory support at ICU admission was classified as room air, nasal oxygen, reservoir-mask oxygen, continuous positive airway pressure or high-flow oxygen, or invasive mechanical ventilation and was included as a categorical baseline covariate. Subsequent escalation of respiratory support, including intubation during follow-up, was not included as a predictor in the primary analysis.

All patients received standard of care according to the national COVID-19 treatment algorithm issued by the Turkish Ministry of Health, unless contraindicated. Standard treatment included acetylsalicylic acid, favipiravir, enoxaparin, and dexamethasone in recommended doses.

The initiation of immunomodulatory therapy was not protocol-driven. Decisions to administer anakinra or tocilizumab were made at the discretion of the attending ICU physician based on clinical deterioration, progression of respiratory failure, and elevated inflammatory markers suggestive of hyperinflammation. Because both agents were used off-label for COVID-19 during the study period, treatment required formal approval through the centralized Ministry of Health off-label use authorization system. Following approval, administration depended on drug availability. Tocilizumab was administered intravenously at a recorded dose of 400 or 800 mg. Anakinra was administered subcutaneously in 100-mg doses, with dosing frequency and treatment duration determined by the treating physician rather than fixed.

Hospital admission defined study time zero, with each comparison indexed at the start of the relevant decision day. Treatment classification was based solely on the action taken that day and never on treatment received subsequently. Patients who died before day 2 were not eligible for the sequential analysis; patients who died after entering the analysis but before receiving an immunomodulator contributed only to control/defer risk sets while alive, hospitalized, and untreated. Mortality was assessed through hospital day 28 and through the final in-hospital outcome.

The calendar date of immunomodulatory therapy initiation relative to ICU admission was recorded. For the primary analysis, treatment exposure was defined as the first valid initiation of anakinra or tocilizumab during hospital days 2–10. At the start of each hospital day, patients who remained alive and hospitalized and had not previously initiated either agent constituted that day’s risk set. Each eligible person-day was classified as anakinra initiation, tocilizumab initiation, or control/defer. The control/defer action was time-dependent and was not equivalent to the original patient-level control group; patients who subsequently initiated anakinra or tocilizumab could therefore contribute control/defer person-time on earlier eligible days.

Patients were excluded from the primary sequential analysis if they had incomplete prespecified baseline covariate data, did not meet the chronology or day-2 risk-set requirements, initiated anakinra or tocilizumab before hospital day 2, had an invalid treatment date, or initiated treatment on or after the final recorded outcome. The outcomes were 28-day in-hospital mortality, defined as death during the index hospitalization on or before hospital day 28, and overall in-hospital mortality, defined as death at any time during the index hospitalization before discharge. Patients discharged alive before day 28 were classified as not having a 28-day in-hospital death; post-discharge vital status was unavailable. All included patients had a recorded in-hospital death or discharge, so there was no censoring in the primary binary-outcome analyses. Original patient-level treatment groups were retained only for descriptive baseline reporting and were distinct from the daily treatment actions used in the sequential analysis.

The Harran University Ethics Committee approved this retrospective review of historical medical records on 30 June 2025 (HRÜ/2512.35) and waived the requirement for informed consent. Records for patients admitted between August 2020 and February 2022 were extracted from the electronic medical record between 7 July and 7 August 2025.

## Statistics

Baseline characteristics were summarized according to the original patient-level treatment groups as median [interquartile range] for continuous variables and number (percentage) for categorical variables. Missing values were reported explicitly, no imputation was performed, and the primary analysis was restricted to records with complete data for all prespecified covariates. Baseline differences were described using the maximum pairwise absolute standardized mean difference (SMD) across the three original groups; baseline hypothesis-test P values were not calculated because these comparisons were descriptive.

The adjustment set for the revised analysis was specified before comparative effect estimation based on clinical relevance, availability before the treatment decision, and usable baseline data coverage; no univariable screening or automated stepwise selection was used. The primary analysis evaluated the baseline-adjusted sequential association between first initiation of anakinra or tocilizumab and mortality during hospital days 2–10. On each decision day, patients who were alive, remained hospitalized, and had not previously initiated either treatment constituted the risk set. The daily actions were initiation of anakinra, initiation of tocilizumab, or control/defer. Control/defer represented a time-indexed analytical action and was not equivalent to the original untreated patient group. Only the first valid calendar-date treatment initiation was considered.

Treatment-action probabilities were estimated using a pooled ridge-penalized multinomial model including decision day, age, sex, CT Fleischner total score, kidney-disease category, structured nonrenal/nonmalignant comorbidity burden, admission respiratory-support severity, and log_2_-transformed baseline lymphocyte count, CRP, creatinine, and D-dimer. Generalized overlap weights were subsequently calibrated using an outcome-blind procedure and normalized to a mean of 1. No matching, caliper, treatment-probability-based discarding, weight truncation, or winsorization was used. Because the treatment model was multinomial, model adequacy was assessed using convergence, pairwise covariate balance across all three actions, weight distributions, and action-specific effective sample size rather than a single binary c-statistic.

Prespecified validity criteria included convergence of the propensity, calibration, and outcome models; an effective sample size of at least 10 for each action; and a maximum calibrated pairwise absolute SMD of 0.20 or less. Associations with 28-day in-hospital mortality and overall in-hospital mortality were estimated using weighted marginal modified-Poisson generalized estimating equations that included decision day and used robust standard errors clustered by patient. Model-standardized marginal risks and risk ratios (RRs) with 95% confidence intervals (CIs) were calculated. Two-sided P values were adjusted using the Holm procedure across the three pairwise comparisons separately within each mortality endpoint.

**Fig 1:**
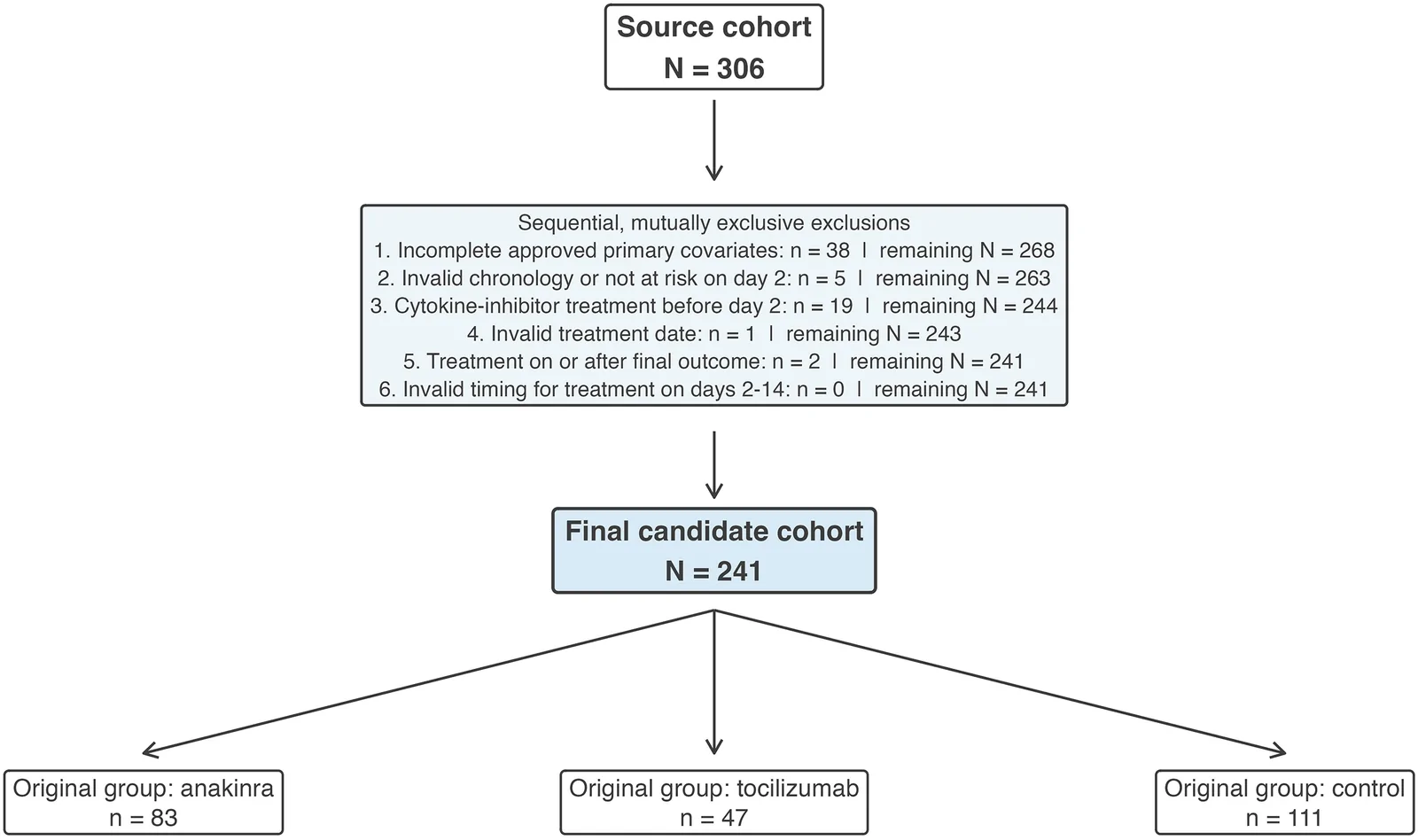
Cohort eligibility flow from 306 source records to 241 included patients. Eligibility criteria were applied sequentially, making exclusion counts mutually exclusive. The final analytic cohort contained 241 patients. Original patient-level treatment groups were used for descriptive baseline reporting only; daily actions were defined separately.

No values were imputed. The primary analysis was restricted to patients with complete data for the prespecified covariates; variable-specific missingness and the complete eligibility waterfall are reported in Fig 1. A prespecified complete-case sensitivity analysis additionally included baseline ferritin. Analyses were performed locally using R version 4.6.0, principally with glmnet version 5.0 and geepack version 1.3.13. R-code development and validation, statistical table and figure preparation, and language editing were assisted by OpenAI Codex. The authors reviewed and verified all outputs and take full responsibility for the manuscript.

## Results

### Study population

Of 306 source records, 38 were excluded because of incomplete prespecified baseline covariates, 5 because of invalid chronology or absence from the day-2 risk set, 19 because cytokine-inhibitor treatment had been initiated before hospital day 2, 1 because of an invalid treatment date, and 2 because treatment occurred on or after the final recorded outcome. No additional records were excluded because of invalid treatment timing during days 2–14. The final analytic cohort comprised 241 patients, including 83 in the original anakinra group, 47 in the original tocilizumab group, and 111 in the original control group (Fig 1). Missing or unusable baseline values occurred for CT Fleischner score (15/306; 4.9%), structured comorbidity burden (13/306; 4.2%), D-dimer (10/306; 3.3%), CRP (4/306; 1.3%), creatinine (4/306; 1.3%), and lymphocyte count (1/306; 0.3%). Because some records had more than one incomplete variable, these categories were not mutually exclusive. Overall, 38 of 306 records (12.4%) were excluded because of incomplete primary covariates.

The median time from admission to the final in-hospital outcome was 11 days (IQR, 6–17). By hospital day 28, 148 patients (61.4%) had died in hospital, 82 (34.0%) had been discharged alive, and 11 (4.6%) remained hospitalized. Of those remaining hospitalized, 10 subsequently died and one was discharged alive; overall, 158 patients (65.6%) died in hospital and 83 (34.4%) were discharged alive.

### Baseline characteristics

The median age was 65 years (IQR, 55–74), and 146 patients (60.6%) were male. At admission, 161 patients (66.8%) required reservoir-mask oxygen, 42 (17.4%) required continuous positive airway pressure or high-flow oxygen, and 5 (2.1%) required invasive mechanical ventilation. The median computed tomography Fleischner total score was 22 points (IQR, 14–30) (Table 1).

**Table 1:** Baseline characteristics of the candidate cohort according to original patient-level treatment group.

Characteristic & category	Overall, N = 241	Anakinra, n = 83	Tocilizumab, n = 47	Control, n = 111	Missing, n	Maximum unweighted pairwise absolute SMD
**A. Demographics**						
**Age, years**	65.0 [55.0, 74.0]	67.0 [55.0, 75.0]	63.0 [52.0, 72.0]	65.0 [57.0, 75.0]	0	0,393
**Sex: Male, n (%)**	146 (60.6%)	44 (53.0%)	33 (70.2%)	69 (62.2%)	0	0,359
**B. Baseline COVID-19 severity**						
**Room air, n (%)**	1 (0.4%)	0 (0.0%)	0 (0.0%)	1 (0.9%)	0	0,135
**Nasal oxygen, n (%)**	32 (13.3%)	3 (3.6%)	5 (10.6%)	24 (21.6%)		0,563
**Reservoir-mask oxygen, n (%)**	161 (66.8%)	62 (74.7%)	38 (80.9%)	61 (55.0%)		0,577
**CPAP/high-flow oxygen, n (%)**	42 (17.4%)	18 (21.7%)	4 (8.5%)	20 (18.0%)		0,374
**Invasive mechanical ventilation, n (%)**	5 (2.1%)	0 (0.0%)	0 (0.0%)	5 (4.5%)		0,307
**CT Fleischner total score, points**	22.0 [14.0, 30.0]	23.0 [14.0, 28.0]	24.0 [18.0, 30.0]	20.0 [14.0, 32.0]	0	0,176
**C. Kidney disease**						
**No documented kidney disease, n (%)**	202 (83.8%)	73 (88.0%)	39 (83.0%)	90 (81.1%)	0	0,191
**Chronic kidney disease, n (%)**	17 (7.1%)	0 (0.0%)	2 (4.3%)	15 (13.5%)		0,559
**End-stage kidney disease, n (%)**	12 (5.0%)	6 (7.2%)	2 (4.3%)	4 (3.6%)		0,161
**Renal transplant, n (%)**	4 (1.7%)	2 (2.4%)	1 (2.1%)	1 (0.9%)		0,118
**Unknown, n (%)**	6 (2.5%)	2 (2.4%)	3 (6.4%)	1 (0.9%)		0,296
**D. Nonrenal and nonmalignant comorbidity**						
**Structured nonrenal/nonmalignant comorbidity burden, conditions**	1.0 [0.0, 2.0]	1.0 [0.0, 2.0]	1.0 [0.0, 2.0]	1.0 [0.0, 2.0]	0	0,116
**Hypertension, n (%)**	106 (44.0%)	29 (34.9%)	22 (46.8%)	55 (49.5%)	0	0,299
**Diabetes mellitus, n (%)**	78 (32.4%)	28 (33.7%)	15 (31.9%)	35 (31.5%)	0	0,047
**Coronary artery disease, n (%)**	34 (14.1%)	11 (13.3%)	8 (17.0%)	15 (13.5%)	0	0,105
**Chronic obstructive pulmonary disease, n (%)**	12 (5.0%)	2 (2.4%)	3 (6.4%)	7 (6.3%)	0	0,195
**Asthma, n (%)**	13 (5.4%)	7 (8.4%)	2 (4.3%)	4 (3.6%)	0	0,204
**Rheumatoid arthritis, n (%)**	6 (2.5%)	3 (3.6%)	0 (0.0%)	3 (2.7%)	0	0,274
**Anxiety, n (%)**	7 (2.9%)	3 (3.6%)	0 (0.0%)	4 (3.6%)	0	0,274
**E. Baseline hematological profile**						
**Leukocyte count**	11740.0 [8780, 15750]	12720.0 [9860, 16490]	13880.0 [10270, 19740]	9960 [7850, 13770]	0	0,666
**Neutrophil count**	10325.0 [7695, 14350]	11300 [8570, 15260]	12665.0 [8830, 18300]	8520 [6060, 13010]	1	0,745
**Lymphocyte count**	690.0 [450, 1010]	620.0 [400, 940]	670.0 [510, 1080]	730 [460, 1040]	0	0,212
**Platelet count**	238000 [180000, 301000]	253000 [198000, 323000]	225000 [167000, 284000]	237000 [161000, 305000]	0	0,368
**Hemoglobin (g/dL)**	12.8 [10.8, 14.2]	12.4 [10.8, 14.2]	12.8 [11.1, 14.3]	12.8 [10.8, 14.0]	0	0,232
**F. Baseline inflammatory and infectious markers**						
**CRP (mg/L)**	117.0 [69.0, 190.0]	114.0 [71.0, 188.0]	128.0 [69.0, 190.0]	112.0 [64.0, 201.0]	0	0,059
**Ferritin (ng/mL)**	744 [433, 1320]	801 [427, 1428]	934 [608, 1371]	655 [386, 1132]	5	0,054
**Procalcitonin**	0.3 [0.1, 0.7]	0.3 [0.1, 0.9]	0.3 [0.1, 0.5]	0.2 [0.1, 0.7]	1	0,218
**G. Kidney function and metabolic profile**						
**Urea (mg/dL)**	52.0 [37.0, 75.0]	54.0 [37.0, 82.0]	54.0 [41.0, 74.0]	49.0 [36.0, 75.0]	0	0,049
**Creatinine (mg/dL)**	0.9 [0.7, 1.4]	0.9 [0.7, 1.4]	0.9 [0.8, 1.4]	0.9 [0.7, 1.4]	0	0,165
**Glucose (mg/dL)**	179 [129, 231]	180 [131, 246]	195 [146, 240]	174 [128, 217]	0	0,161
**Sodium (mmol/L)**	139 [136, 142]	140 [136, 143]	137 [135, 140]	138.0 [135, 141]	0	0,483
**Potassium (mmol/L)**	4.5 [4.1, 4.9]	4.4 [4.0, 4.9]	4.6 [4.2, 5.0]	4.4 [4.2, 4.8]	0	0,276
**H. Liver and tissue-injury markers**						
**AST**	33.0 [24.0, 49.0]	32.0 [20.0, 44.0]	36.0 [25.0, 50.0]	35.0 [24.0, 52.0]	0	0,215
**ALT**	26.0 [14.0, 45.0]	23.0 [14.0, 44.0]	34.0 [16.0, 55.0]	25.0 [12.0, 43.0]	0	0,267
**LDH**	516 [387, 667]	519 [413, 656]	635 [499, 954]	386 [288, 514]	170	1,123
**Troponin**	16.0 [8.0, 41.0]	18.0 [10.0, 47.0]	13.0 [7.3, 34.0]	15.0 [7.0, 42.0]	8	0,197
**I. Coagulation and thromboinflammatory profile**						
**D-dimer**	1.4 [0.6, 4.1]	1.7 [0.7, 7.3]	2.3 [0.8, 7.9]	0.9 [0.4, 2.9]	0	0,625
**INR**	1.1 [1.0, 1.2]	1.2 [1.1, 1.3]	1.1 [1.0, 1.2]	1.1 [1.0, 1.2]	0	0,293
**Fibrinogen, unit not verified**	5.2 [4.0, 6.6]	5.3 [4.0, 6.6]	5.0 [3.7, 6.9]	5.1 [4.2, 6.3]	10	0,037
**J. Other verified baseline severity markers**						
**Lactate (mmol/L)**	2.0 [1.7, 2.6]	2.1 [1.7, 2.6]	2.0 [1.9, 2.4]	2.0 [1.7, 2.6]	8	0,129

The original patient-level treatment groups showed appreciable baseline heterogeneity. Notable unweighted baseline imbalances included lactate dehydrogenase, neutrophil and leukocyte counts, D-dimer, respiratory-support categories, chronic kidney disease, sodium, and age. These original groups were used only for descriptive baseline reporting and were distinct from the time-indexed daily treatment actions evaluated in the sequential analysis (Table 1).

### Sequential risk sets and treatment initiation

**Fig 2:**
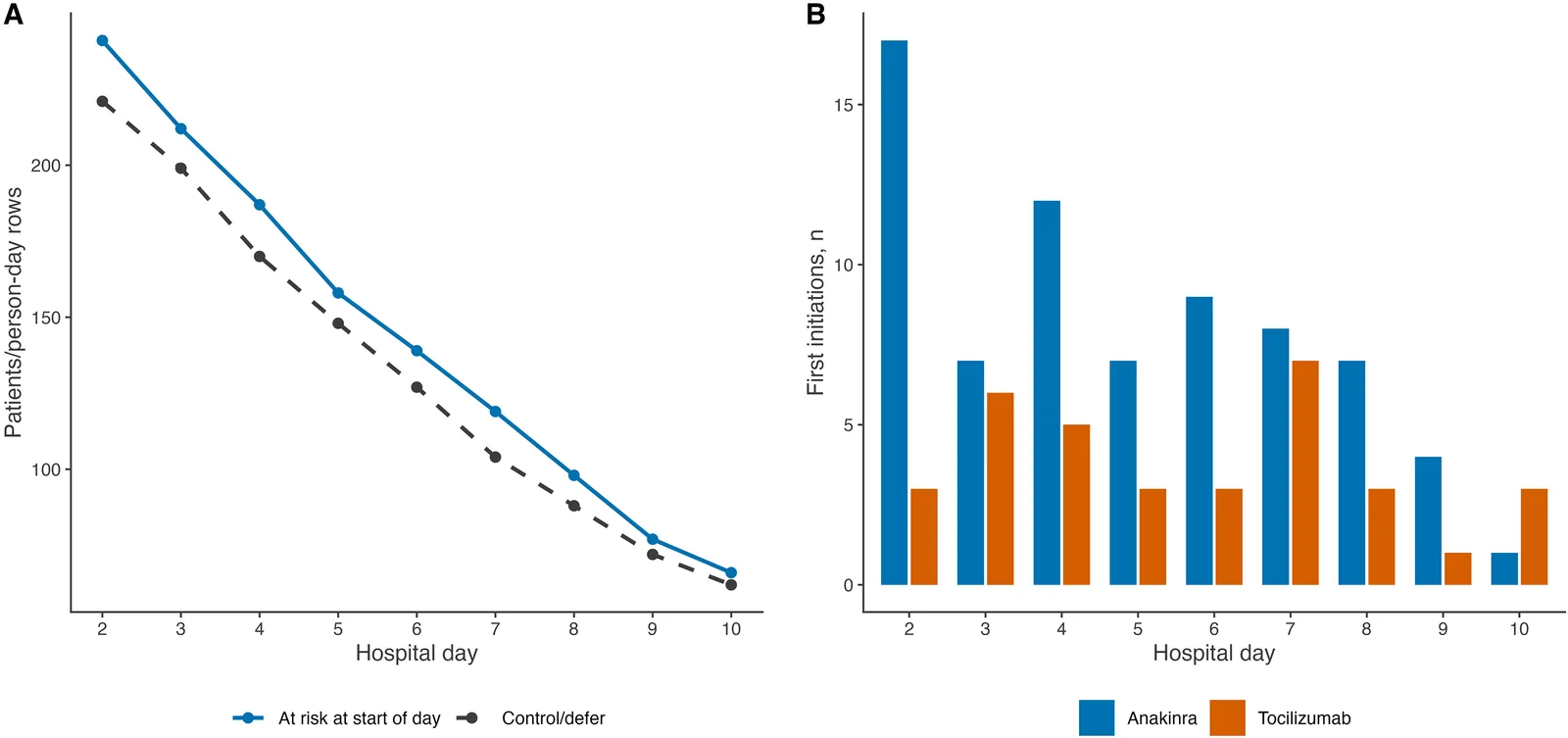
Sequential risk sets and first cytokine-inhibitor initiation during hospital days 2-10. Panel A shows at-risk and control/defer person-day counts. Panel B shows first valid calendar-date initiation counts. Control/defer was a time-indexed analytical action and was not equivalent to the original patient-level control group; it could include untreated person-time before later treatment initiation. The primary window contributed 1,297 person-days: 1,191 control/defer, 72 anakinra, and 34 tocilizumab actions.

The sequential analysis evaluated daily treatment decisions during hospital days 2–10. The number of patients at risk decreased from 241 on day 2–66 on day 10. Across the decision window, 1,297 person-day observations were available, comprising 1,191 control/defer actions, 72 first anakinra initiations, and 34 first tocilizumab initiations. Anakinra initiation occurred most frequently on day 2 (n = 17), whereas tocilizumab initiation occurred most frequently on day 7 (n = 7) (Table 2 and Fig 2). Control/defer represented a daily analytical action and could include untreated person-time contributed before subsequent initiation of anakinra or tocilizumab.

**Table 2:** Sequential risk sets and first cytokine-inhibitor initiation during hospital days 2-10.

Hospital day	At risk at start of day	Control/defer action	Anakinra initiation	Tocilizumab initiation	Daily action total	Anakinra initiation, %	Tocilizumab initiation, %	Cumulative anakinra initiation	Cumulative tocilizumab initiation
**2**	241	221	17	3	241	7,1%	1,2%	17	3
**3**	212	199	7	6	212	3,3%	2,8%	24	9
**4**	187	170	12	5	187	6,4%	2,7%	36	14
**5**	158	148	7	3	158	4,4%	1,9%	43	17
**6**	139	127	9	3	139	6,5%	2,2%	52	20
**7**	119	104	8	7	119	6,7%	5,9%	60	27
**8**	98	88	7	3	98	7,1%	3,1%	67	30
**9**	77	72	4	1	77	5,2%	1,3%	71	31
**10**	66	62	1	3	66	1,5%	4,5%	72	34
**Days 2–10 total**	1.297	1.191	72	34	1.297	5,6%	2,6%	72	34

### Weighting, calibration, and covariate balance

**Fig 3:**
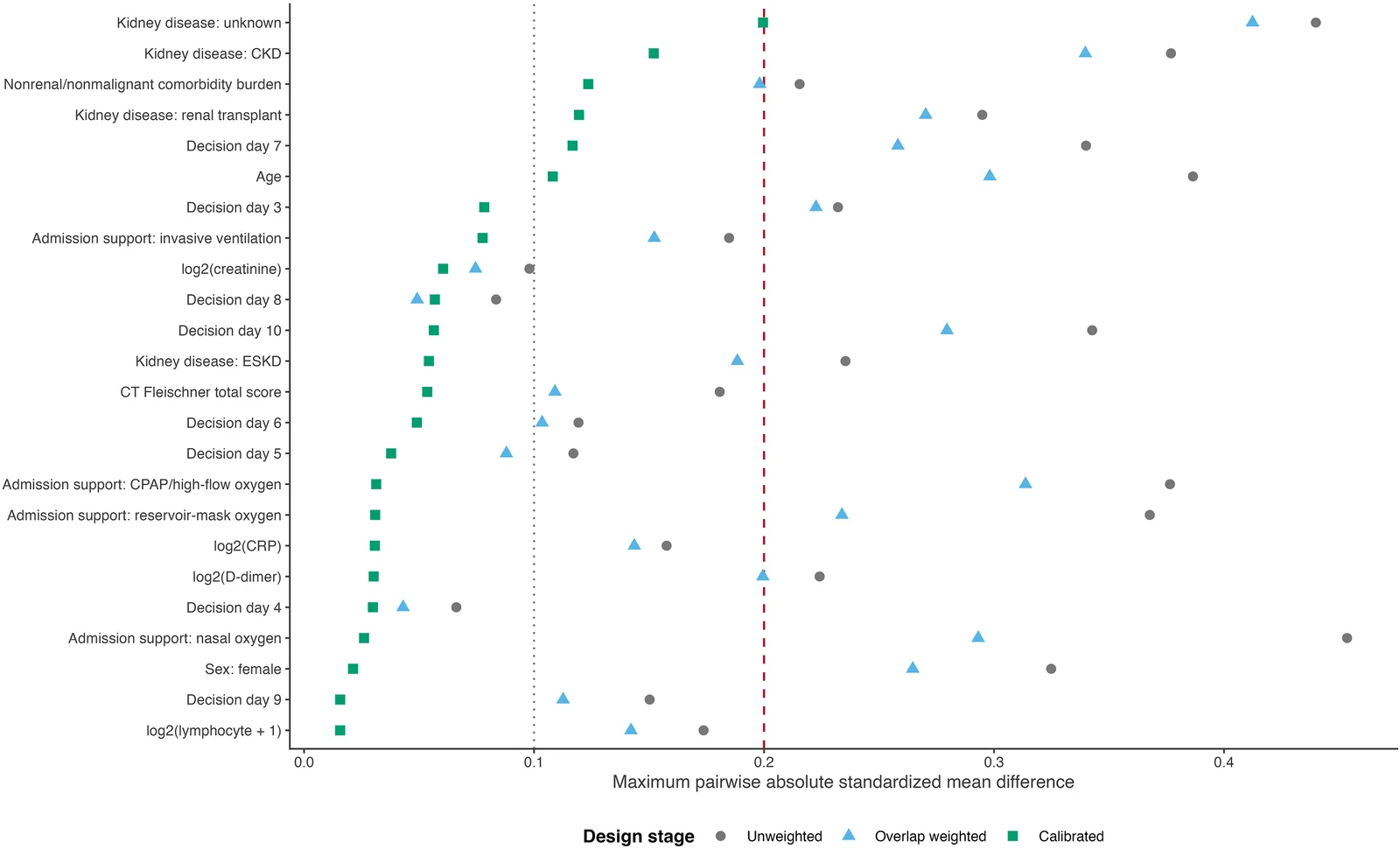
Love plot of covariate balance before weighting, after generalized overlap weighting, and after calibration. Each point is the worst pairwise absolute SMD for the indicated model-matrix component. Reference lines mark 0.10 and the prespecified 0.20 threshold. The maximum calibrated SMD was 0.1996; the balance criterion was met narrowly and does not establish elimination of confounding.

Before weighting within the sequential daily risk sets, the maximum pairwise absolute standardized mean difference (SMD) was 0.454. Generalized overlap weighting reduced this value to 0.412, and subsequent outcome-blind calibration reduced it further to 0.1996, narrowly meeting the prespecified balance criterion of ≤0.20 (Table 3 and Fig 3).

**Table 3:** Propensity-weighting diagnostics, covariate balance, and effective sample size.

Diagnostic	Control/defer	Anakinra	Tocilizumab	Overall or worst	Prespecified criterion
**Person-day action rows**	1191	72	34	1297	–
**Effective sample size**	997.5	51.8	18.7	18.7	>=10 for every action
**Median calibrated weight**	0.58	3.65	6.51	0.60	Descriptive
**Maximum calibrated weight**	1.58	16.41	35.20	35.20	Descriptive
**Maximum pairwise absolute SMD: Unweighted**	–	–	–	0.454	Descriptive
**Maximum pairwise absolute SMD: Overlap**	–	–	–	0.412	Descriptive
**Maximum pairwise absolute SMD: Calibrated**	–	–	–	0.1996	<=0.20

The effective sample sizes after calibration were 997.5 for control/defer, 51.8 for anakinra initiation, and 18.7 for tocilizumab initiation. Median calibrated weights were 0.58, 3.65, and 6.51, respectively, and the corresponding maximum weights were 1.58, 16.41, and 35.20. Although the prespecified effective-sample-size and balance thresholds were met, the small effective sample size and large maximum weight for tocilizumab indicated limited effective support for that action (Table 3 and S1 Fig). Weights were normalized to a mean of 1 and were not truncated or winsorized.

### Twenty-eight-day in-hospital mortality

**Fig 4:**
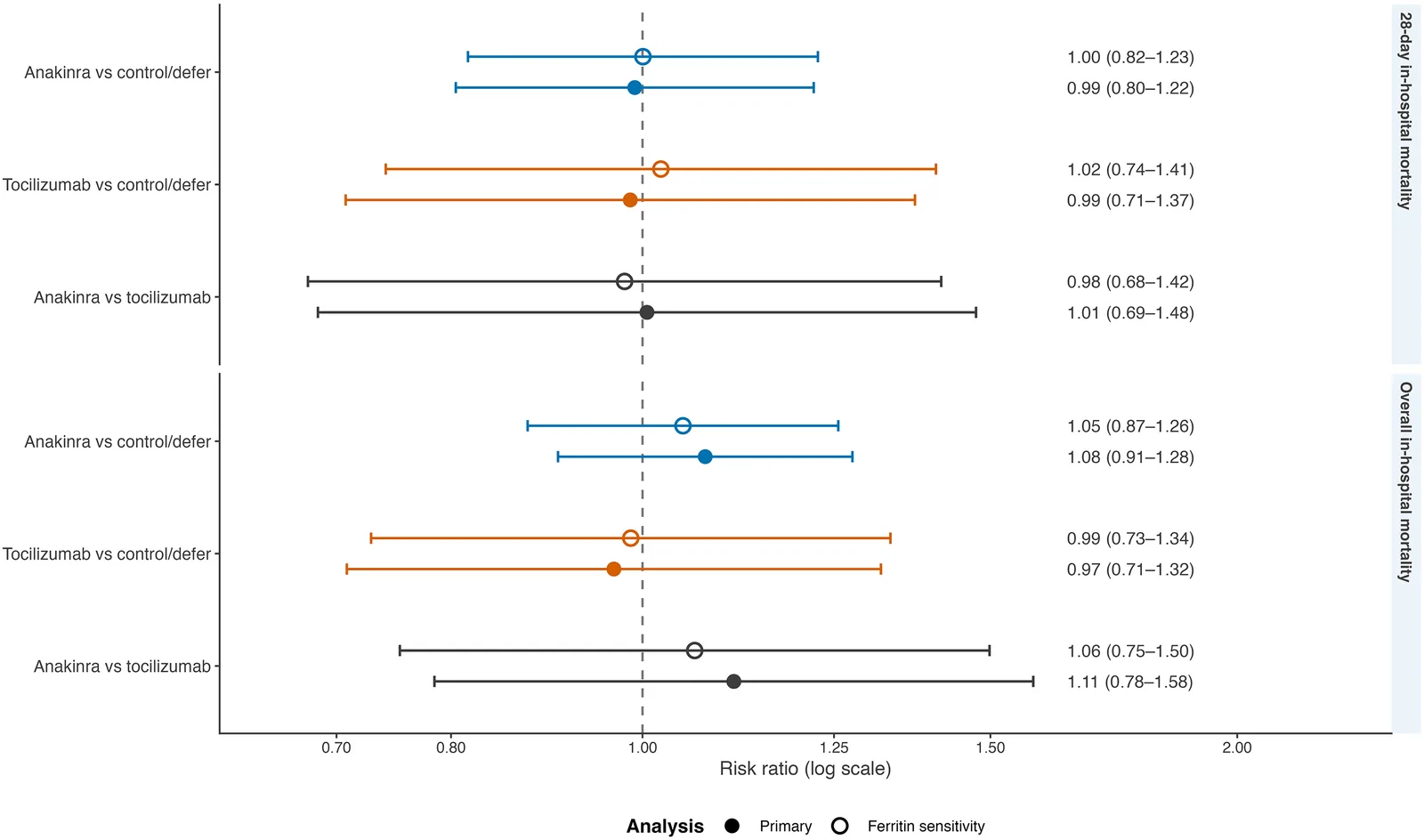
Primary and ferritin-sensitivity adjusted mortality associations. Points and horizontal lines are risk ratios and robust 95% confidence intervals from the baseline-adjusted sequential daily modified-Poisson analysis. Filled symbols are primary estimates and open symbols are ferritin-sensitivity estimates. All confidence intervals included 1. Estimates are observational associations and do not establish equivalence or definitive causal effects.

Detailed model-standardized risks are presented in Table 4 and Fig 4. Neither anakinra nor tocilizumab initiation was clearly associated with 28-day in-hospital mortality relative to control/defer (anakinra: RR, 0.99; 95% CI, 0.80–1.22; P = 0.932; tocilizumab: RR, 0.99; 95% CI, 0.71–1.37; P = 0.933). The head-to-head comparison was also inconclusive (RR, 1.01; 95% CI, 0.69–1.48; P = 0.979). The Holm-adjusted P value was 1.000 for each comparison.

**Table 4:** Baseline-adjusted associations of daily treatment initiation with 28-day and overall in-hospital mortality.

Endpoint	Comparison	Weighted risk, comparator action	Weighted risk, reference action	Risk ratio	95% CI	Two-sided P value	Holm-adjusted P value	Ferritin-sensitivity risk ratio	Ferritin-sensitivity 95% CI
**28-day in-hospital mortality**	Anakinra vs control/defer	61,6%	62,2%	0,99	0.80-1.22	0,932	1,000	1,00	0.82-1.23
**28-day in-hospital mortality**	Tocilizumab vs control/defer	61,3%	62,2%	0,99	0.71-1.37	0,933	1,000	1,02	0.74-1.41
**28-day in-hospital mortality**	Anakinra vs tocilizumab	61,6%	61,3%	1,01	0.69-1.48	0,979	1,000	0,98	0.68-1.42
**Overall in-hospital mortality**	Anakinra vs control/defer	71,7%	66,6%	1,08	0.91-1.28	0,404	1,000	1,05	0.87-1.26
**Overall in-hospital mortality**	Tocilizumab vs control/defer	64,4%	66,6%	0,97	0.71-1.32	0,834	1,000	0,99	0.73-1.34
**Overall in-hospital mortality**	Anakinra vs tocilizumab	71,7%	64,4%	1,11	0.78-1.58	0,550	1,000	1,06	0.75-1.50

### Overall in-hospital mortality

Detailed model-standardized risks are presented in Table 4 and Fig 4. Compared with control/defer, the RR was 1.08 (95% CI, 0.91–1.28; P = 0.404) for anakinra initiation and 0.97 (95% CI, 0.71–1.32; P = 0.834) for tocilizumab initiation. The RR comparing anakinra with tocilizumab was 1.11 (95% CI, 0.78–1.58; P = 0.550). Holm-adjusted P values were 1.000 for all three comparisons.

### Sensitivity and descriptive analyses

The prespecified complete-case sensitivity analysis additionally adjusting for baseline ferritin yielded estimates consistent with the primary analysis. For 28-day in-hospital mortality, the RRs were 1.00 (95% CI, 0.82–1.23) for anakinra versus control/defer, 1.02 (95% CI, 0.74–1.41) for tocilizumab versus control/defer, and 0.98 (95% CI, 0.68–1.42) for anakinra versus tocilizumab. For overall in-hospital mortality, the corresponding RRs were 1.05 (95% CI, 0.87–1.26), 0.99 (95% CI, 0.73–1.34), and 1.06 (95% CI, 0.75–1.50), respectively (Table 4 and Fig 4).

The descriptive weighted cumulative in-hospital mortality curves showed broadly similar trajectories across the three daily actions and were not used for the primary inferential comparisons (S2 Fig). All 95% confidence intervals from the primary and ferritin-sensitivity analyses included 1. Accordingly, the analyses did not identify a clear association between initiation of anakinra or tocilizumab and either mortality endpoint. The imprecision of the estimates, limited support for tocilizumab initiation, narrowly achieved balance criterion, and possibility of residual or unmeasured confounding preclude conclusions of treatment equivalence or definitive causal effects.

## Discussion

In this single-center ICU cohort of critically ill patients with COVID-19, baseline-adjusted sequential analyses did not identify a clear association between initiation of anakinra or tocilizumab during hospital days 2–10 and either 28-day or overall in-hospital mortality. Model-standardized risks were similar across the daily treatment actions, and all primary risk-ratio confidence intervals included the null value; estimates were materially unchanged after additional adjustment for baseline ferritin. These findings should not, however, be interpreted as evidence of treatment equivalence or absence of a clinically important effect. Covariate balance met the prespecified criterion only narrowly after outcome-blind calibration, effective support for tocilizumab initiation was limited, and the observational design remains susceptible to residual and unmeasured confounding. Accordingly, the results indicate no demonstrable mortality benefit or harm within the precision and support of the present analysis rather than definitive ineffectiveness of either cytokine-targeted therapy.

The literature on IL-6 and IL-1 blockade in COVID-19 reports heterogeneous findings across study designs and clinical settings. In a French multicenter retrospective cohort, Arcani et al. found no significant difference in 28-day mortality between tocilizumab and anakinra after propensity matching (29.1% vs 30.4%) [15]. In contrast, Küçükşahin et al., in a single-center matched ward cohort, reported lower ICU admission and mortality rates with anakinra compared with tocilizumab; however, higher corticosteroid exposure in the anakinra group represents an important confounder [16].

Randomized evidence indicates that treatment effects vary by agent, disease stage, and patient selection. RECOVERY demonstrated improved outcomes with tocilizumab in hospitalized patients with hypoxia and systemic inflammation, whereas final REMAP-CAP findings supported tocilizumab and sarilumab [17,18]. In contrast, SAVE-MORE reported benefit from early, soluble urokinase plasminogen activator receptor–guided anakinra in selected hospitalized patients before critical illness [19]. These differences in population, treatment timing, and selection criteria limit direct comparison with the present ICU cohort. In ICU-based retrospective analyses, Abdelnaby et al. and Klopfenstein et al. reported reduced mortality and/or mechanical ventilation requirements with tocilizumab, whereas a prospective cohort study from Mexico found no mortality difference compared with standard therapy [20–22]. Similarly, the randomized trial by Salama et al. showed that tocilizumab reduced progression to mechanical ventilation or death among hospitalized patients not yet ventilated, without significantly improving overall survival [23]. A multicenter retrospective study with the primary outcomes of intubation or death revealed that tocilizumab was associated with a lower odds ratio of intubation, while no significant difference was observed in terms of mortality [24].

With respect to IL-1 blockade, Huet et al. reported reduced ICU admission and mortality with anakinra, whereas De la Calle et al. observed no prognostic improvement in patients unresponsive to tocilizumab [25,26]

This study has several limitations. Its retrospective, single-center design limits generalizability. Treatment initiation was clinician-directed and influenced by clinical deterioration, inflammatory activity, off-label approval, and drug availability. Although the sequential daily risk-set design prevented preinitiation survival from being attributed to treated actions, it could not eliminate confounding by indication, treatment access, or incompletely measured time-varying severity. Treatment use was also strongly separated by calendar period, and admission calendar time was not included in the primary adjustment set; evolving clinical practice, vaccination, and circulating variants may therefore have confounded the estimates. Admission respiratory-support category was included, but several important prognostic factors—including PaO_2_/FiO_2_ ratio, SOFA, APACHE II or SAPS II scores, baseline vasopressor or renal-replacement therapy, corticosteroid dose, vaccination status, coinfection, immunosuppressive therapy, and time from symptom onset—were unavailable or insufficiently reliable for adjustment.

Covariate balance met the prespecified criterion only narrowly after calibration (maximum absolute SMD, 0.1996), and effective support for tocilizumab was limited (ESS, 18.7; maximum weight, 35.2), reducing precision. Complete-case analysis may have introduced selection bias, and vital status after discharge was unavailable. Accordingly, the null-spanning confidence intervals cannot establish equivalence or exclude a clinically important benefit or harm; the findings should be interpreted as adjusted associations rather than causal effects. Methodological strengths include calendar-dated treatment initiation within sequential daily risk sets, prespecified covariate adjustment, outcome-blind calibration, explicit balance and support diagnostics, patient-clustered robust inference, and a ferritin-adjusted sensitivity analysis.

## Conclusions

In this single-center retrospective ICU cohort, initiation of anakinra or tocilizumab during hospital days 2–10 was not clearly associated with 28-day or overall in-hospital mortality after adjustment for prespecified baseline covariates. Ferritin-adjusted estimates were similar, but imprecision, limited effective support for tocilizumab initiation, and potential residual and time-varying confounding preclude conclusions of equivalence or definitive causal effects. Further prospective studies are needed.

## Supporting information

S1 Fig Calibrated weight distributions and calibration balance-support trade-off. Panel A shows calibrated weights by daily action. Panel B shows the outcome-blind calibration grid. The selected solution had minimum action ESS 18.7 and maximum absolute SMD 0.1996. Weights were not truncated, winsorized, or manually altered.
(JPG)

S2 Fig Descriptive weighted cumulative in-hospital mortality curves. (JPG)
